# GWAMAR: Genome-wide assessment of mutations associated with drug resistance in bacteria

**DOI:** 10.1186/1471-2164-15-S10-S10

**Published:** 2014-12-12

**Authors:** Michal Wozniak, Jerzy Tiuryn, Limsoon Wong

**Affiliations:** 1 Faculty of Mathematics, Informatics and Mechanics, University of Warsaw, Warsaw, Poland; 2 School of Computing, National University of Singapore, Singapore

**Keywords:** drug resistance, bacteria, Mycobacterium tuberculosis, comparative genomics, compensatory mutations

## Abstract

**Background:**

Development of drug resistance in bacteria causes antibiotic therapies to be less effective and more costly. Moreover, our understanding of the process remains incomplete. One promising approach to improve our understanding of how resistance is being acquired is to use whole-genome comparative approaches for detection of drug resistance-associated mutations.

**Results:**

We present *GWAMAR*, a tool we have developed for detecting of drug resistance-associated mutations in bacteria through comparative analysis of whole-genome sequences. The pipeline of GWAMAR comprises several steps. First, for a set of closely related bacterial genomes, it employs eCAMBer to identify homologous gene families. Second, based on multiple alignments of the gene families, it identifies mutations among the strains of interest. Third, it calculates several statistics to identify which mutations are the most associated with drug resistance.

**Conclusions:**

Based on our analysis of two large datasets retrieved from publicly available data for *M. tuberculosis*, we identified a set of novel putative drug resistance-associated mutations. As a part of this work, we present also an application of our tool to detect putative compensatory mutations.

## Background

The development of drug resistance in bacteria makes antibiotics less effective and increases the costs of therapies. This problem has drawn the attention of major health organizations such as WHO (World Health Organization), ECDC (European Centre for Disease Prevention and Control) and CDC (Centers for Disease Control and Prevention) which monitor and report the epidemics of drug resistance in the world [1,2].

Over a few decades of research on drug resistance, several drug-resistance mechanisms have been discovered such as alteration of a drug's binding site, reduced accumulation of a drug, and specialized enzymes to degrade drug molecules [3]. These mechanisms may be acquired either through chromosomal mutations or horizontal gene transfer.

There are many mutations reported in various studies as associated with drug resistance mechanisms. However, the information is spread throughout the literature and not easy to access. One attempt to collect the information on genetic features associated with drug resistance into a database is the Antibiotic Drug Resistance Database (ARDB) [4]. However, this database focuses on genes associated with drug resistance rather than particular point mutations within them. Another species-specific database of drug resistance-associated mutations in *M. tuberculosis *is the Tuberculosis Drug Resistance Mutation Database (TBDReaMDB) [5]. This database provides detailed information on a set of 1230 associations between drugs and point mutations. Furthermore, it distinguishes a subset of *high-confidence *mutations which were often reported in literature.

The process of acquisition of drug resistance is often associated with some additional cost, called fitness, which reduces the general viability of the bacteria [6,7]. However, this effect may be reversed completely or partially by secondary mutations, called compensatory mutations [7,8]. Consequently, the evolutionary process of acquiring multiple drug resistance happens in a sequence of steps rather than simultaneously [9-11]. Thus, in order to better understand the process, it is important to identify not only the primary mutations responsible directly for drug resistance, but also those secondary mutations allowing for gradual accumulation of drug resistance.

Since the number of bacterial genomes being sequenced and publicly available sequencing data grows rapidly [12], it opens new possibilities for using large-scale computational approaches for identifying drug resistance-associated mutations.

In our previous work, we presented an approach to use whole-genome comparative approach for identification of drug resistance-associated mutations [13]. We proposed a new computational scoring scheme, called *weighted support*, which uses phylogenetic information for identifying the drug resistance-associated mutations. In order to test our approach, we used publicly available sequencing data for 100 strains of *Staphylococcus aureus *and collected phenotype data from over 70 articles. Our experiment suggested that weighted support outperforms other standard measures such as mutual information and odds ratio.

In the current work, we present GWAMAR, a tool for identifying of drug resistance-associated mutations based on comparative analysis of whole-genome sequences of closely related bacterial strains. This tool is designed as a pipeline. It first employs eCAMBer, our previously published tool [14], to identify point mutations among the set of considered genomes. These mutations constitute the genotype data. Then, GWAMAR tries to find the associations between the input phenotype and genotype data by computing several statistical scores. As a part of this work, we also designed a new statistical score, viz *tree-generalized hypergeometric score *(TGH).

The rational for incorporating phylogenetic information into TGH and weighted support is based on our observation that subtrees of the phylogenetic tree very often correspond to geographic locations. Since drug resistance mutations are subject to evolutionary pressure caused by the drug treatment they should be independent of geographic location and therefore be more widely distributed over the tree, as opposed to mutations driven by other environmental factors which tend to rather concentrate in small subtrees.

In order to test our approach, we run GWAMAR on two large datasets for *M. tuberculosis*. The first dataset is prepared for the set of 173 strains with genome sequences and annotations publicly available in the PATRIC database [15]. And for this set of strains, we collected drug resistance information from over 20 publications. The genotype and phenotype data for the second dataset comes from the *M. tuberculosis *Drug Resistance Directed Sequencing Database.

## Methods

We present details of GWAMAR in this section, including the problem setting, the preprocessing of input data and the computation of the association scores between the drug resistance phenotypes and point mutations.

### Problem setting

We consider a set  of closely related bacterial genomes. Typically, this is a set of strains within the same species of bacteria.

Then, we represent the available drug resistance information as a set of *drug-resistance profiles *, where each drug resistance profile r∈R is represented as a vector:

r:S→{S,I,R,?}.

Here, *S, I, R *denote that a given strain is known to be drug susceptible, intermediate-resistant, or resistant, respectively. Using question mark ${}^{‵}{?}^{\prime }$ we indicate that the drug resistance status of a strain is unknown. We call a drug resistance profile *complete *if it does not contain question marks.

Analogously, we represent the genotype data as a set of mutations , where each mutation m∈M is represented as a triple (*g, p, v*), where *g,p,v *denote the gene family identifier, the position of the mutation in its corresponding multiple alignment, and the *mutation profile*, respectively. The mutation profile is represented as a vector:

v:S→∑∪{?}.

Here Σ denotes the set of amino acides (for protein-coding genes) or nucleotides (for promoters and rRNA coding genes). We also assume that Σ contains the gap '-' symbol. Using question mark ${}^{‵}{?}^{\prime }$ we indicate that the corresponding amino acid or nucleotide is unknown. Analogously, we call a mutation profile *complete *if it does not contain question marks.

It should be noted that potentially multiple mutations at different positions in the genome may have identical mutation profiles. Moreover, it may happen that multiple mutations may correspond to the same set of mutated strains. In that situation the mutations would essentially carry the same information about the profiles. Thus, we also introduce an auxiliary concept called *binary mutation profile*. Let r∈S denote the reference strain and s∈S denote any strain. Then, for a given *mutation profile v *its corresponding binary mutation profile is defined as follows:

$$b\left(s\right)=\left\{\begin{array}{cc} ? & i\text{f}\;\text{v(s)}=?\\ 0 & \text{if}\;\text{v(s)}=\text{v(r)}\\ 1 & \text{otherwise} \end{array}\right)$$

Analogous to mutation profiles, we call a binary mutation profile *complete *if it does not contain question marks.

Finally, we define the aim of the tool as: To produce an ordered list of associations between the phenotype and genotype data (represented as drug resistance and mutation profiles) such that the top-scored associations are the most likely to be real.

### The pipeline of GWAMAR

**Figure 1:**
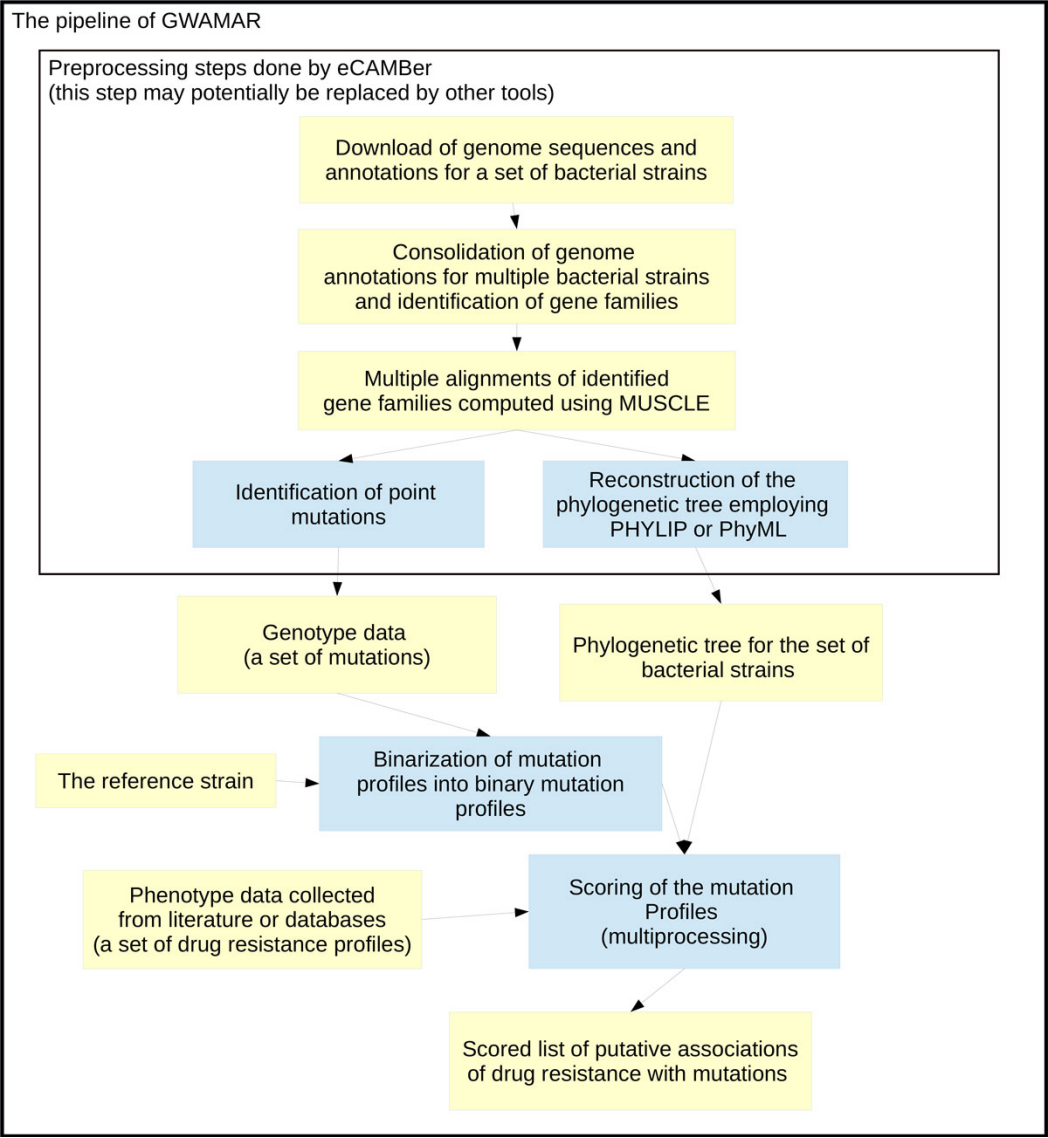
**Schema of the GWAMAR pipeline**. For a set of considered bacterial strains, the input data for GWAMAR consists of (i) a set of mutations; (ii) a set of drug resistance profiles; and (iii) phylogenetic tree for the set of bacterial strains. Typically the set of mutation profiles is generated using eCAMBer, which is able to download the genome sequences and annotations for the set of bacterial strains, identify point mutations based on multiple alignments, and reconstruct the phylogenetic tree of the considered bacterial strains. The first step of GWAMAR is to compute binary mutation profiles for all point mutations. This step significantly reduces the number of genetic profiles considered. Finally, GWAMAR implements several statistical scores to associate drug resistance profiles with mutation profiles. These include: mutual information, odds ratio, hypergeometric test, weighted support and tree-generalized hypergeometric score (TGH). As a result, we obtain ordered lists of drug resistance associations, where the top scored associations are the most likely to be real.

Figure 1 illustrates the overall design of the tool. Data preprocessing for GWAMAR consists of several steps which may be performed by our previously developed tool, eCAMBer [14]. These preprocessing steps comprise:

• download genome sequences of multiple bacterial strains,

• unification of gene annotations,

• identification of homologous gene families,

• multiple alignments of the gene families (employing MUSCLE),

• reconstruction of the phylogenetic tree (employing PHYLIP),

• identification of point mutations based on the multiple alignments.

The input drug-resistance profiles, typically, are collected from the articles or databases which provide drug-resistance information for the strains of interest. The set of identified point mutations, the set of drug-resistance profiles and and the phylogenetic tree constitute the input for GWAMAR.

In the next step, GWAMAR computes binary mutation profiles for each mutation profile. This step significantly reduces the number of genetic profiles to be scored. Finally, GWAMAR computes several statistical scores to associate drug-resistance profiles to the mutation profiles, including mutual information, odds ratio, hypergeometric test, weighted support (which is our previously published approach [13]), and the tree-generalized hypergeometric score (our new approach here).

#### Tree-generalized hypergeometric score

As a part of this work we also introduce a new association score, called tree-generalized hypergeometric score (*TGH *). This score is a modification of the *CCTSWEEP *score introduced in the paper [16]. In this section, we consider a subset of strains *S *for which a given drug-resistance profile *r *and a binary mutation profile *b *are complete, i.e. do not contain question marks. Moreover, we assume that *r *does not contain any intermediate-resistant strains. In all our computational experiments we transform the intermediate-resistant strains into resistant strains.

In order to present the formal definition of TGH, we first define an auxiliary concept called *coloring*. For a given tree *T *, we call a subset *c *of its nodes a *coloring*, if it satisfies the following two conditions:

• each path from a leaf to the root contains at most one node from *c*,

• each internal node in *c *has a sibling node which does not belong to *c*.

Here we also introduce a function *L *which, for each node *ω*, returns the set of descendants of the node, including the node itself. We say these nodes are visible from *ω*. Additionally, the function *L *applied to a coloring *c *returns the union of all nodes visible from nodes in *c*.

Let *C_T _*denote the set of all colorings of *T *. Then, for each complete drug-resistance profile *r *there exists exactly one coloring *ĉ *such that the set of leaves visible from *ĉ *equals the set of drug-resistant nodes in *r*. We say this coloring is induced by the drug-resistance profile. Analogously, for each complete binary mutation profile *b *there is exactly one induced coloring $\bar{c}$.

Intuitively, the coloring induced by a given complete drug-resistance profile will contain the set of nodes in which drug-resistance was acquired (assuming a model in which drug-resistance cannot be reversed). Analogously, the coloring induced by a given binary mutation profile will contain the set of nodes in which the mutation was acquired.

**Figure 2:**
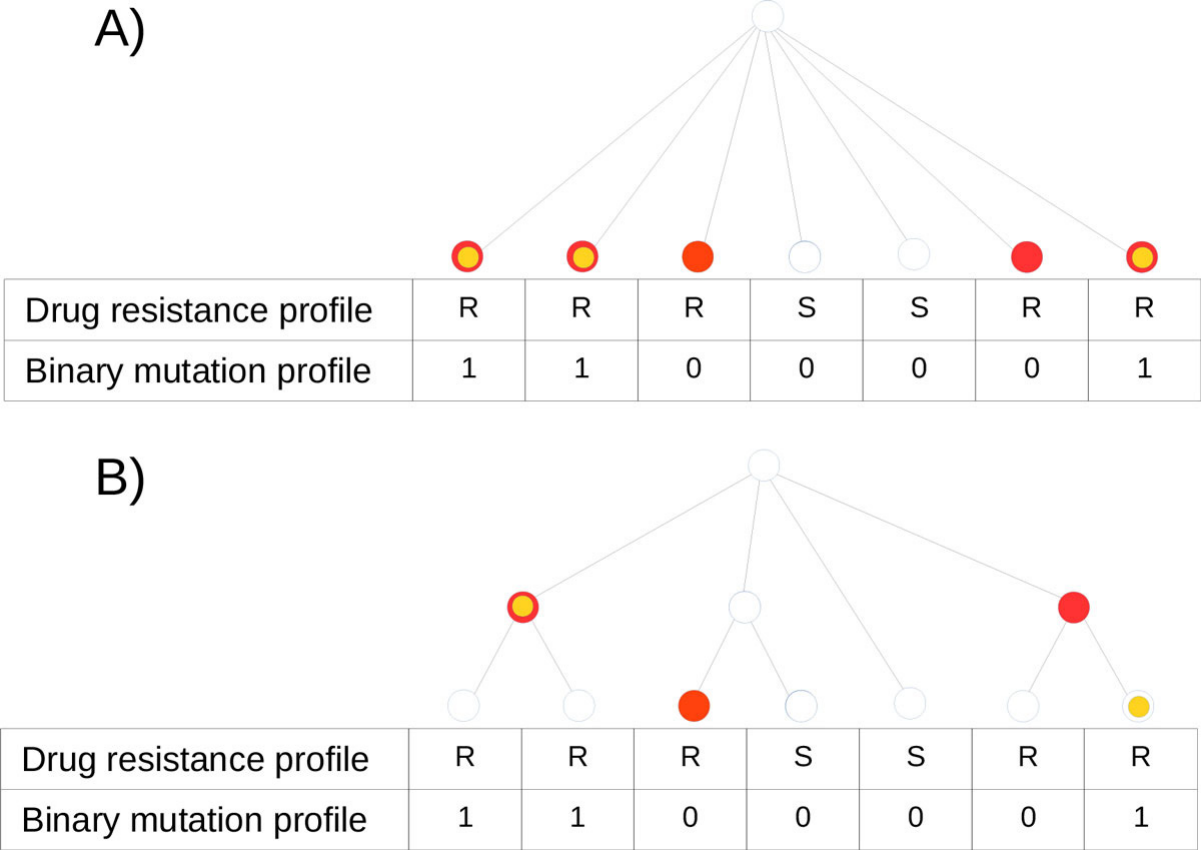
**Example colorings for the TGH score**. (A) an example of coloring *ĉ *induced by a given drug-resistance profile (large red nodes) and coloring $\bar{c}$ induced by a given binary mutation profile (small orange nodes) for a flat tree. In this example $|ĉ|=5,|\bar{c}|=3$ and $|L\left(ĉ\right)\cap \bar{c}|=3$. (B) another example of colorings *ĉ *and $\bar{c}$ induced by the same pair of profiles but for a different tree. In this example $|ĉ|=3,|\bar{c}|=2$ and $|L\left(ĉ\right)\cap \bar{c}|=2$*|*

Figure 2 (A) presents an example of colorings induced by a given drug-resistance profile (large red nodes) and a given binary mutation profile (small orange nodes) for a flat tree. In that situation the colorings may be interpreted as independent drawing of balls as in the standard hypergeometric distribution model. Knowing this property of TGH we proposed its name as it generalizes the standard hypergeometric test in the case of a flat tree. Figure 2 (B) presents another example of colorings induced by the same pair of profiles, but for a tree which is not flat. In this model the dependencies between different strains are captured by the topology of the tree.

Let us now assume we want to compute the TGH score for a pair of complete drug-resistance profile *r *and complete binary mutation profile *b*. Let us additionally assume that the size of coloring $\bar{c}$ induced by *b *equals *n*. Morover, let the number of nodes in coloring $\bar{c}$ visible from the coloring *ĉ *equals *k*. This value can be interpreted as the number of times the considered mutation was acquired not earlier than the resistance was acquired.

Now, let *V_T_*(*n*) denote the number of colorings of size *n*:

$$V_{T}\left(n\right)=\#\left\{c\in C_{T}:|c|=n\right\}$$

*V_T_*(*n*) may be interpreted as the total number of binary mutation profiles for which the induced coloring is of the same size as for $\bar{c}$.

Then, let *B_T, ĉ_*(*k, n*) denote the number of colorings of size *n*, such that exactly *k *nodes of that coloring are visible from nodes of coloring *ĉ*.

$$B_{T,ĉ}\left(k,n\right)=\#\left\{c\in C_{T}:|L\left(ĉ\right)\cap c|=k\;\text{and}\;|c|=n\right\}$$

Here, the value *B_T, ĉ_*(*k, n*) may be interpreted as the number of binary mutation profiles such that their induced coloring has *n *elements, out of which *k *is visible from the nodes in *ĉ*.

Finally, for the complete drug-resistance profile *r *and complete binary mutation profile *b*, which induce colorings *ĉ *and $\bar{c}$, respectively, we define the TGH score as follows:

$$H_{T}\left(r,b\right)=-\text{log}\left(\frac{\sum_{i=k}^{n}B_{T,ĉ}\left(i,n\right)}{V_{T}\left(n\right)}\right)$$

Here, we take the negative logarithm to have consistent property for all considered scoring methods, such that the higher the score the more likely drug-resistance profile *r *is associated with binary mutation profile *b*.

### Time complexity

Let *D *denote the number of drug-resistance profiles considered. Additionally, let *N *denote the number of considered strains and *M *denote the number of binary mutation profiles. Finally, let *K *denote the maximal number of children of an internal node in the tree. Then, the time complexity of the algorithms we implemented to compute the hypergeometic score, the mutual information, odds ratio, and weighted support is *O*(*D N M *). In order to compute TGH, we implement a dynamic programing algorithm which computes the values *B_ω,ĉ_*(*k, n*) for each internal node *ω, k *and *n*. This strategy gives an algorithm with complexity *O*(*D*·*N ^K−^*^1^·*N*^2 ^+*D*·*N*·*M *). For the brevity of the presentation we skip details of the algorithm.

## Results and discussion

We now present and discuss results we obtained applying GWAMAR on the two datasets we prepared for *M. tuberculosis*.

In all the following analyzes we use the set of mutations classified as *high-confidence *mutations in Tuberculosis Drug Resistance Mutation Database (TBDReaMDB) as our gold standard [5]. In total the database contains 88 of high-confidence associations spanning 27 genes and six drugs or drug families: Fluoroquinolones, Ethambutol, Isoniazid, Pyrazinamide, Rifampicin and Streptomycin.

### Mtu173 case study

The first case study is based on the set of 173 fully sequenced strains of *M. tuberculosis *with publicly available data.

The preprocessing steps of preparing the genotype data were performed using eCAMBer, our previously published tool to support comparative analysis of multiple bacterial strains [13].

In particular, first, we used eCAMBer to download the genome sequences and annotations from the PATRIC database [15]. Next, we applied eCAMBer to unify the genome annotations of protein-coding genes and to identify the clusters of genes with high sequence similarity. Then, for the subset of 4379 such identified gene clusters with genes present in at least 90% of the strains, we computed multiple alignments using MUSCLE [17]. The multiple alignments were computed for amino-acid sequences of protein-coding genes, as well as nucleotide sequences of their promoter regions, and rRNA genes. Finally, based on the computed multiple alignments, we identified 118913 non-synonymous point mutations. The set of identified point mutations constituted the input genotype data for GWAMAR.

The input phenotype data was collected from over 20 publications issued together with the fully sequenced genomes. Additional file 1 shows the drug-resistance status of the strains retrieved from literature together with references. Based on the drug-resistance information collected for Ciprofloxacin and Ofloxacin, we introduced a new drug-resistance profile for Fluoroquinolones. A strain is categorized as susceptible to Fluoroquinolones if it was susceptible to at least one of the drugs, but not resistant to any of them. Similarly, a strain was categorized as resistant to Fluoroquinolones if it was resistant to at least one of the drugs, but not susceptible to any of them. If none of the cases was satisfied for a strain, then the drug-resistance status of the strain was categorized as unknown. We restrict further analysis to the set of six drugs or drug families: Fluoroquinolones, Ethambutol, Isoniazid, Pyrazinamide, Rifampicin and Streptomycin.

The input phylogenetic tree was reconstructed using the maximum-likelihood approach implemented in the PHYLIP package. As an input for the tool we used the set of all the identified mutations concatenated into one multiple alignment file. Additional file 2 presents the reconstructed phylogenetic tree.

Table 1 presents the list of top 20 associations ordered according to TGH score. In the set of 20 top-scoring associations, 15 are present in the TBDReaMDB database and 13 of them are categorized as high-confidence mutations. A closer look at the mutations which are not present in TBDReaMDB revealed that some of them can be supported by literature. In particular, mutation E504G/D in embB has recently been reported as associated with resistance to Ethambutol [18]. Similarly, the mutation T539I has already been associated with resistance to Fluoroquinolones [19].

**Table 1:** 20 top-scoring associations between drug-resistance profiles and point mutations in the case study on 173 fully sequenced *M.tuberculosis *strains.

drug name	gene id	gene name	mutation	all	**h.c**.	TGH
Fluoroquinolones	Rv0006	gyrA	D94G/A/H/N/Y	Y	Y	14.1843430424
Isoniazid	Rv1908c	katG	S315T/G/N	Y	Y	9.04507605888
Rifampicin	Rv0667	rpoB	S450L	Y	Y	8.60191917013
Streptomycin	Rv0682	rpsL	K43R	Y	Y	8.32303955124
Ethambutol	Rv3795	embB	M306I/V/L	Y	Y	8.24966301883
Isoniazid	Rv1483	fabG1	C-15T	Y	Y	5.8445976648
Rifampicin	Rv0667	rpoB	D435F/V/Y/G/A	Y	Y	5.0402225732
Streptomycin	Rv0682	rpsL	K88R/M	Y	Y	4.16354931535
Ethambutol	Rv3795	embB	E504G/D	N	N	3.33103155053
Pyrazinamide	Rv2043c	pncA	W68L	Y	Y	2.7080502011
Pyrazinamide	Rv2043c	pncA	H51P	Y	Y	2.7080502011
Rifampicin	Rv0667	rpoB	H445D/Y/R	Y	Y	2.52993515037
Streptomycin	Rvnr01	rrs	G1108C	N	N	1.71691080314
Ethambutol	Rv3795	embB	D1024N	Y	N	1.68763546921
Ethambutol	Rv3795	embB	D869G	N	N	1.68763546921
Ethambutol	Rv3795	embB	A505T	N	N	1.68763546921
Fluoroquinolones	Rv0005	gyrB	N538T	Y	Y	1.68478734968
Fluoroquinolones	Rv0006	gyrA	S91P	Y	Y	1.68478734968
Fluoroquinolones	Rv0005	gyrB	T539I	N	N	1.68478734968
Streptomycin	Rvnr01	rrs	A1401G	Y	N	1.28846347057

Each row corresponds to one association, whereas the consecutive columns describe: drug name, gene identifier, gene name, mutation, association presence in the TBDReaMDB database, status indicating if the association is categorized as high confidence in TBDReaMDB, TGH score.

Literature search did not provide us any additional support for the remaining three mutations (A505T in embB, D869G in embB and G1108C in rrs), which haven't been reported in TBDReaMDB. These mutations may potentially be false positives or real drug-resistance-associated mutations.

### Mtu_broad case study

The second case study, *mtu_broad*, is based on the data available in the Broad Institute database http://www.broadinstitute.org/annotation/genome/mtb_drug_resistance.1/. This database contains provides sequencing data and drug-resistance information for 1398 strains of *M. tuberculosis*. However, it should be noted that only genes of interest were sequenced. Table 2 presents the list of 28 sequenced genes for each strain. Additionally 12 promoter sequences were sequenced. In total, this database contains 1069 mutations (non-synonymous amino-acid changes or nucleotide changes in promoters).

**Table 2:** List of sequenced genes and promoters available in the *mtu_broad *dataset.

gene id	gene name	description	promoter sequenced?
Rv0005	gyrB	DNA gyrase subunit B	yes
Rv0006	gyrA	DNA gyrase subunit A	yes
Rv0341	iniB	isoniazid inductible gene protein	yes
Rv0342	iniA	isoniazid inductible gene protein	yes
Rv0343	iniC	isoniazid inductible gene protein	yes
Rv0667	rpoB	DNA-directed RNA polymerase beta chain	yes
Rv0682	rpsL	30S ribosomal protein S12	yes
Rv1483	fabG1	3-oxoacyl-[acyl-carrier protein] reductase	yes
Rv1484	inhA	NADH-dependent enoyl-[acyl-carrier-protein] reductase	yes
Rv1694	tlyA	cytotoxin--haemolysin	no
Rv1854c	ndh	NADH dehydrogenase	yes
Rv1908c	katG	catalase-peroxidase-peroxynitritase T	no
Rv2043c	pncA	pyrazinamidase/nicotinamidas	yes
Rv2245	kasA	3-oxoacyl-[acyl-carrier protein] synthase 1	no
Rv2427Ac	oxyR'	hypothetical protein	no
Rv2428	ahpC	alkyl hydroperoxide reductase C protein	yes
Rv2764c	thyA	thymidylate synthase	yes
Rv2764c	ddl	D-alanine-D-alanine ligase ddlA	no
Rv3423c	alr	alanine racemase	no
Rv3793	embC	membrane indolylacetylinositol arabinosyltransferase	yes
Rv3794	embA	membrane indolylacetylinositol arabinosyltransferase	yes
Rv3795	embB	membrane indolylacetylinositol arabinosyltransferase	yes
Rv3854c	ethA	monooxygenase	yes
Rv3919c	gid	glucose-inhibited division protein B	yes
Rvnr01	rrs	ribosomal RNA 16S	no
Rvnr02	rrl	ribosomal RNA 23S	no

Similar to the previous case study, based on the drug-resistance information available in the database for Ciprofloxacin, Ofloxacin, Levofloxacin and Moxifloxacin, we introduced a new drug-resistance profile for Fluoroquinolones. A strain was categorized as susceptible to Fluoroquinolones if it was susceptible to at least one of the drugs, but not resistant to any of them. Similarly, a strain was categorized as resistant to Fluoroquinolones if it was resistant to at least one of the drugs, but not susceptible to any of them. If none of the cases was satisfied for a strain, then the drug-resistance status of the strain was categorized as unknown. We restrict further analysis to the set of six drugs or drug families: Fluoroquinolones, Ethambutol, Isoniazid, Pyrazinamide, Rifampicin and Streptomycin.

Similarly, as in the previous case study, the phylogenetic tree was reconstructed using the maximum-likelihood approach implemented in the PHYLIP package. As an input for the tool we used the set of all available mutations concatenated into one multiple alignment file.

Table 3 presents the list of the top 20 associations ordered according to TGH score. In the set of 20 top-scoring associations, 19 are present in TBDReaMDB and 15 of them are categorized as high-confidence mutations.

**Table 3:** 20 top-scoring associations between drug-resistance profiles and point mutations in the case study for 1398 partially sequenced *M.tuberculosis *strains.

drug name	gene id	gene name	mutation	all	**h.c**.	TGH
Fluoroquinolones	Rv0006	gyrA	D94A/G/N/Y/H	Y	Y	129.754964792
Fluoroquinolones	Rv0006	gyrA	A90G/V	Y	Y	41.8967753922
Streptomycin	Rv0682	rpsL	K43R	Y	Y	31.005838239
Isoniazid	Rv1908c	katG	S315T/S/G/N/I/R	Y	Y	27.1918713598
Ethambutol	Rv3795	embB	Q497P/R/K/H	Y	Y	17.1681425414
Streptomycin	Rv0682	rpsL	K88T/R/Q/M	Y	Y	16.2806822989
Fluoroquinolones	Rv0005	gyrB	N538K/T/D/S	Y	Y	12.6368065275
Rifampicin	Rv0667	rpoB	H445P/D/R/Y/L/N/Q	Y	Y	12.627849397
Streptomycin	Rvnr01	rrs	A1401G	Y	N	9.60726487825
Pyrazinamide	Rv2043c	pncA	T135P/A	Y	N	9.35766011848
Streptomycin	Rvnr01	rrs	A514C	Y	Y	8.96892262877
Rifampicin	Rv0667	rpoB	D435Y/V/H/G/A/N	Y	Y	7.63431166207
Fluoroquinolones	Rv0006	gyrA	S91P	Y	Y	7.57935978224
Pyrazinamide	Rv2043c	pncA	T-11C/G	Y	Y	6.76727069266
Ethambutol	Rv3795	embB	G406S/D/A/C	Y	Y	6.32500852932
Fluoroquinolones	Rv0006	gyrA	D89G/N	N	N	6.26814578901
Pyrazinamide	Rv2043c	pncA	L120P/R	Y	N	6.11085770664
Streptomycin	Rvnr01	rrs	C517T	Y	Y	5.16411345885
Ethambutol	Rv3795	embB	D328Y/G/H	Y	N	5.07901609928
Pyrazinamide	Rv2043c	pncA	V139G/A/M/L	Y	Y	5.05727324518

This dataset is provided by The Broad Institute. Each row corresponds to one association, whereas the consecutive columns describe: drug name, gene identifier, gene name, mutation, association presence in the TBDReaMDB database, status indicating if the association is categorized as high confidence in TBDReaMDB, TGH score.

A closer look at the mutation D89G/N, which is not present in TBDReaMDB, reveals that the mutation has recently been associated with resistance to Fluoroquinolones [20].

The set of associations provides some additional support for the four mutations which were present in TBDReaMDB, but not categorized as high-confidence.

### Assessment of accuracy

Here we use the two datasets described above to assess the accuracy of the various association scores, viz: mutual information, odds ratio, hypergeometric score, weighted support and TGH. The CCTSWEEP software might contain some bugs and did not produce correct output. Its authors had not responded to our queries in time. So we omitted it from our experiments.

As our gold standard we use the set of 88 drug-resistance associations classified as *high-confidence *in the Tuberculosis Drug Resistance Mutation Database (TBDReaMDB) [5].

In both case studies, as the set of positives, we assume the subset of high-confidence mutations present in TBDReaMDB, which are also present in the genotype data. This is the set of mutations which may be potentially detected using the available datasets. There are 39 and 75 of such associations for the *mtu173 *and *mtu_broad *datasets, respectively. The set of negatives is constructed by random sampling from the whole set of identified putative associations except for the associations which are classified as positives. The number of sampled negatives equals the total number of mutations present in genes which has at least one high-confidence mutation. We use this approach in order to significantly reduce the probability of classifying as negatives associations which are real but not present in the database.

**Figure 3:**
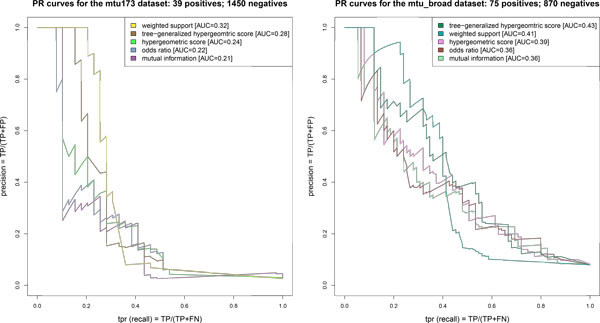
**Comparison of accuracy**. Precision-recall curves for comparison of different association scores implemented in GWAMAR. Left panel presents results for the *mtu173 *dataset; right for the *mtu_broad *dataset. Numbers present in the square brackets display the Area Under the Curve (AUC) for the scores. In both case studies tree-aware statistics (weighted support and TGH) achieve better performance the the tree-ignorant statistics.

Figure 3 presents precision and recall curves for different association scores. Consistently, results on both datasets suggest that tree-aware scores outperform tree-ignorant scores.

### Rifampicin resistance and compensatory mutations

It is commonly known that Rifampicin resistance in *M. tuberculosis *gets acquired by point mutations within the RRDR region which corresponds to the Rifampicin binding spot [21]. However, these mutations are often associated with deleterious effect on bacteria fitness [22]. This effect may be potentially reversed by compensatory mutations. Recently, three new papers have been released, focusing on identifying putative compensatory mutations within rpoA, rpoB and rpoC genes [23-25].

**Figure 4:**
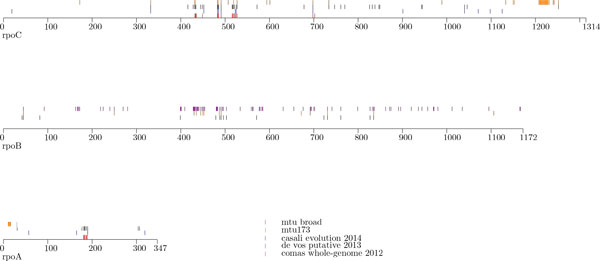
**Compensatory mutations**. Distribution of putative compensatory mutations with the *rpoA, rpoB *and *rpoC *genes. Position of each mutation is indicated by a vertical line.

Figure 4 presents the distribution of the putative compensatory mutations identified in these recent studies, put together with mutations identified based on our two case studies.

Interestingly, several mutations identified by our approach have also been reported in at least one of the papers [23-25].

• rpoA: G31S/A

• rpoB: P45S/L, L731P, E761D, R827C, H835P/R

• rpoC: G332R/S, V431M, G433C/S, V483G/A, W484G, I491T/V, L527V, N698K, A734V

Additional file 3 contains the detailed table with the complete list of putative compensatory mutations.

## Conclusion

In this work we presented GWAMAR, a tool we have developed for identifying of drug-resistance associated mutations based on comparative analysis of whole-genome sequences in bacterial strains.

The tool is designed as an automatic pipeline which employs eCAMBer, our previously published tool [14], for preprocessing of data. This preprocessing includes: (i) download of genome sequences and gene annotations, (ii) unification of gene annotations among the set of considered strains, (iii) identification of gene families, (iv) computation of multiple alignments and identification of point mutations which constitute the input genotype data.

GWAMAR implements various statistical methods--such as mutual information, odds ratio, hypergeometric test and weighted support [13]-- to associate the drug-resistance phenotypes with point mutations. As a part of this work, we also present *tree-generalized hypergeometric score *(TGH) -- a new statistical score.

In order to test our approach, we prepared two datasets for *M. tuberuclosis*. Results of both case studies suggest that tree-aware methods (weighted support and TGH) perform better than methods which do not incorporate phylogenetic information. This result supports also our corollary from our previous paper about weighted support [13]. Employing GWAMAR on the two datasets, we identified novel mutations putatively associated with drug-resistance.

Finally, despite some promising results, the presented tool has some limitations. First, it does not take into account or predict epistatic interactions between mutations. Second, it only takes into account genomic changes ignoring levels of gene expression. Thirdly, it provides putative *in-silico *associations which should be subjected to further investigation in wet lab experiments.

## Availability

The GWAMAR software, input data and results of the case study experiments are available at the website: http://bioputer.mimuw.edu.pl/gwamar.

## Competing interests

The authors declare that they have no competing interests.

## Authors' contributions

All authors contributed to design of the method, analysis of results and writing of the manuscript. MW wrote software and performed experiments. All authors read and approved the final manuscript.

## Supplementary Material

Additional file 1 Drug-resistance information for 173 strains of *M. tuberculosis*. Drug-resistance information collected from literature for 173 strains of *M. tuberculosis*. Click here for file

Additional file 2 Phylogenetic tree for 173 strains of *M. tuberculosis*. Phylogenetic tree reconstructed using the maximum-likelihood method implemented in the PHYLIP for 173 strains of *M. tuberculosis*. Click here for file

Additional file 3 **Putative compensatory mutations**. Complete list of putative compensatory mutations identified in three different studies and our two datasets. Click here for file

## Declarations

Funding for the publication of this paper was provided by Wong's KITHCT chair professorship.

This article has been published as part of *BMC Genomics *Volume 15 Supplement 10, 2014: Proceedings of the 25th International Conference on Genome Informatics (GIW/ISCB-Asia): Genomics. The full contents of the supplement are available online at http://www.biomedcentral.com/bmcgenomics/supplements/15/S10.
