# Supplementary material for: GWAMAR: Genome-wide assessment of mutations associated with drug resistance in bacteria
Source: BMC Genomics. 2014 Dec 12;15(Suppl 10):S10. doi: 10.1186/1471-2164-15-S10-S10 (PMC4304204; doi:10.1186/1471-2164-15-S10-S10)
Supplement: Additional file 1 — Drug-resistance information for 173 strains of M. tuberculosis. Drug-resistance information collected from literature for 173 strains of M. tuberculosis. [file 1471-2164-15-S10-S10-S1.pdf]

**Table 1. Complete collected data on drug resistance.**

| Strain | Streptomycin | Rifampicin | Ethambutol | Isoniazid | Ofloxacin | Kanamycin | Capreomycin | Amikacin | Ethionamide | Pyrazinamide | Cycloserine | Ciprofloxacin | PAS | Rifabutin |
|---|---|---|---|---|---|---|---|---|---|---|---|---|---|---|
| 02 1987 | S [11] | S [11] | S [11] | S [11] | S [11] | S [11] | S [11] | S [11] | S [11] | S [11] | S [11] | S [11] | S [11] | S [11] |
| 210 | | S [13] | S [13] | S [13] | | | | | | | | | | |
| 7199-99 | S [12] | S [12] | S [12] | S [12] | S [12] | S [12] | S [12] | S [12] | S [12] | S [12] | | | S [12] | |
| 94 M4241A | S [11] | S [11] | S [11] | S [11] | S [11] | S [11] | S [11] | S [11] | S [11] | S [11] | S [11] | S [11] | S [11] | S [11] |
| 98-R604 INH-RIF-EM | S [11] | R [11] | R [11] | R [11] | S [11] | S [11] | | | | | | | | |
| BT1 | R [17] | R [17] | R [17] | R [17] | R [17] | R [17] | R [17] | R [17] | R [17] | R [17] | | | | |
| BT2 | R [17] | R [17] | R [17] | R [17] | R [17] | R [17] | R [17] | R [17] | R [17] | R [17] | | | | |
| BTB05-552 | S [14] | S [14] | S [14] | R [14] | S [14] | | | S [14] | | S [14] | | | | |
| BTB05-559 | S [14] | S [14] | S [14] | R [14] | S [14] | | | S [14] | | S [14] | | | | |
| C | S [11] | S [11] | S [11] | S [11] | S [11] | S [11] | S [11] | S [11] | S [11] | S [11] | S [11] | S [11] | S [11] | S [11] |
| CCDC5079 | S [11]S [23] | S [11]S [23] | S [11]S [23] | S [11]S [23] | S [11] | S [11] | S [11] | S [11] | S [11] | S [11] | S [11] | S [11] | S [11] | S [11] |
| CCDC5080 | R [23] | R [23] | R [23] | R [23] | | | | | | | | | | |
| CDC1551 | S [8] | S [8] | S [8] | S [8] | S [8] | S [8] | S [8] | S [8] | S [8] | S [8] | | | | |
| CPHL A | S [11] | S [11] | S [11] | S [11] | S [11] | S [11] | S [11] | S [11] | S [11] | S [11] | S [11] | S [11] | S [11] | S [11] |
| CTRI-2 | S [11]S [1] | S [11]S [1] | S [11]S [1] | S [11]S [1] | S [11]S [1] | S [11] | S [11]S [1] | S [11]S [1] | S [11]S [1] | S [11]S [1] | S [11] | S [11] | S [11] | S [11] |
| CTRI-4 | R [1] | R [1] | R [1] | R [1] | R [1] | | R [1] | R [1] | R [1] | R [1] | | | | |
| EAI OSDD271 | R [18] | R [18] | R [18] | R [18] | | | | | | R [18] | | | R [18] | |
| EAS054 | S [11] | S [11] | S [11] | S [11] | S [11] | S [11] | S [11] | S [11] | S [11] | S [11] | S [11] | S [11] | S [11] | S [11] |
| Erdman | S [11]S [10] | S [11]S [10] | S [11]S [10] | S [11]S [10] | S [11] | S [11]S [10] | S [11]S [10] | S [11]S [10] | S [11]S [10] | S [11]S [10] | S [11]S [10] | S [11] | S [11] | S [11]S [10] |
| FJ05194 | R [7] | R [7] | R [7] | R [7] | R [7] | R [7] | R [7] | | R [7] | | | | | |
| GM 1503 | S [11] | S [11] | S [11] | S [11] | S [11] | S [11] | S [11] | S [11] | S [11] | S [11] | S [11] | S [11] | S [11] | S [11] |
| GuangZ0019 | R [7] | R [7] | R [7] | R [7] | R [7] | R [7] | R [7] | | R [7] | | | | | |
| H37Ra | S [11] | S [11] | S [11] | S [11] | S [11] | S [11] | S [11] | S [11] | S [11] | S [11] | S [11] | S [11] | S [11] | S [11] |
| H37Ra WGS | S [11] | S [11] | S [11] | S [11] | S [11] | S [11] | S [11] | S [11] | S [11] | S [11] | S [11] | S [11] | S [11] | S [11] |
| H37Rv | S [11] | S [11] | S [11] | S [11] | S [11] | S [11] | S [11] | S [11] | S [11] | S [11] | S [11] | S [11] | S [11] | S [11] |
| HKBS1 | S [17] | S [17] | S [17] | S [17] | | | | | | | | | | |
| HN878 | S [3] | S [3] | S [3] | S [3] | S [3] | S [3] | S [3] | S [3] | S [3] | S [3] | S [3] | S [3] | S [3] | S [3] |
| K85 | S [11] | S [11] | S [11] | S [11] | S [11] | S [11] | S [11] | S [11] | S [11] | S [11] | S [11] | S [11] | S [11] | S [11] |
| KZN 1435 | S [11] | R [11] | S [11] | R [11] | S [11] | S [11] | | | | | | | | |
| KZN 4207 | S [11]S [4] | S [11]S [4] | S [11] | S [11]S [4] | S [11]S [4] | S [11]S [4] | S [11] | S [11] | S [11] | S [11] | S [11] | S [11] | S [11] | S [11] |
| KZN 4207 Broad | S [11] | S [11] | S [11] | S [11] | S [11] | S [11] | S [11] | S [11] | S [11] | S [11] | S [11] | S [11] | S [11] | S [11] |
| KZN 605 | R [11] | R [11] | R [11] | R [11] | R [11] | R [11] | | | | | | | | |
| KZN R506 | R [4] | R [4] | | R [4] | R [4] | R [4] | | | | | | | | |
| KZN V2475 | R [4] | R [4] | | R [4] | | | | | | | | | | |
| MTB-476 | S [5] | S [5] | S [5] | S [5] | S [5] | S [5] | S [5] | S [5] | S [5] | S [5] | S [5] | S [5] | S [5] | S [5] |
| MTB-489 | S [5] | S [5] | S [5] | S [5] | S [5] | S [5] | S [5] | S [5] | S [5] | S [5] | S [5] | S [5] | S [5] | S [5] |
| OM-V02 005 | | R [16] | | R [16] | | | | | | | | | | |
| OSDD105 | R [20] | R [20] | R [20] | R [20] | S [20] | S [20] | S [20] | S [20] | S [20] | R [20] | R [20] | | S [20] | |
| OSDD493 | R [19] | R [19] | R [19] | R [19] | R [19] | R [19] | S [19] | S [19] | R [19] | S [19] | S [19] | | S [19] | |
| OSDD515 | R [21] | R [21] | R [21] | R [21] | S [21] | S [21] | R [21] | S [21] | R [21] | R [21] | R [21] | | S [21] | |
| PanR0201 | R [6] | R [6] | S [6] | R [6] | | | | | | | | | | |
| PanR0202 | R [6] | R [6] | R [6] | R [6] | | | | | | | | | | |
| PanR0203 | R [6] | R [6] | R [6] | R [6] | | | | | | | | | | |
| PanR0205 | R [6] | R [6] | R [6] | R [6] | | | | | | | | | | |
| PanR0206 | R [6] | R [6] | S [6] | R [6] | | | | | | | | | | |
| PanR0207 | R [6] | R [6] | S [6] | R [6] | | | | | | | | | | |
| PanR0208 | R [6] | R [6] | S [6] | R [6] | | | | | | | | | | |
| PanR0209 | R [6] | R [6] | R [6] | R [6] | | | | | | | | | | |
| PanR0301 | R [6] | R [6] | S [6] | R [6] | | | | | | | | | | |
| PanR0304 | R [6] | R [6] | R [6] | R [6] | | | | | | | | | | |
| PanR0305 | S [6] | R [6] | S [6] | R [6] | | | | | | | | | | |
| PanR0306 | R [6] | R [6] | R [6] | R [6] | | | | | | | | | | |
| PanR0307 | S [6] | R [6] | S [6] | R [6] | | | | | | | | | | |
| PanR0308 | R [6] | R [6] | R [6] | R [6] | | | | | | | | | | |
| PanR0309 | R [6] | R [6] | S [6] | R [6] | | | | | | | | | | |
| PanR0311 | R [6] | R [6] | S [6] | R [6] | | | | | | | | | | |
| PanR0313 | S [6] | R [6] | S [6] | R [6] | | | | | | | | | | |
| PanR0314 | R [6] | R [6] | S [6] | R [6] | | | | | | | | | | |
| PanR0315 | S [6] | R [6] | S [6] | R [6] | | | | | | | | | | |
| PanR0316 | R [6] | R [6] | S [6] | R [6] | | | | | | | | | | |
| PanR0317 | R [6] | R [6] | S [6] | R [6] | | | | | | | | | | |
| PanR0401 | R [6] | R [6] | R [6] | R [6] | | | | | | | | | | |
| PanR0402 | R [6] | R [6] | S [6] | R [6] | | | | | | | | | | |
| PanR0403 | R [6] | R [6] | S [6] | R [6] | | | | | | | | | | |
| PanR0404 | S [6] | R [6] | S [6] | R [6] | | | | | | | | | | |
| PanR0405 | R [6] | R [6] | R [6] | R [6] | | | | | | | | | | |
| PanR0407 | R [6] | R [6] | S [6] | R [6] | | | | | | | | | | |
| PanR0408 | S [6] | R [6] | S [6] | R [6] | | | | | | | | | | |
| PanR0409 | S [6] | R [6] | S [6] | R [6] | | | | | | | | | | |
| PanR0410 | R [6] | R [6] | S [6] | R [6] | | | | | | | | | | |
| PanR0411 | R [6] | R [6] | S [6] | R [6] | | | | | | | | | | |
| PanR0412 | R [6] | R [6] | S [6] | R [6] | | | | | | | | | | |
| PanR0501 | R [6] | R [6] | S [6] | R [6] | | | | | | | | | | |
| PanR0503 | S [6] | R [6] | S [6] | R [6] | | | | | | | | | | |
| PanR0505 | R [6] | R [6] | R [6] | R [6] | | | | | | | | | | |
| PanR0601 | R [6] | R [6] | S [6] | R [6] | | | | | | | | | | |
| PanR0602 | S [6] | R [6] | S [6] | R [6] | | | | | | | | | | |
| PanR0603 | R [6] | R [6] | R [6] | R [6] | | | | | | | | | | |
| PanR0604 | R [6] | R [6] | R [6] | R [6] | | | | | | | | | | |
| PanR0605 | R [6] | R [6] | S [6] | R [6] | | | | | | | | | | |
| PanR0606 | S [6] | R [6] | S [6] | R [6] | | | | | | | | | | |
| PanR0607 | R [6] | R [6] | S [6] | R [6] | | | | | | | | | | |
| PanR0609 | R [6] | R [6] | S [6] | R [6] | | | | | | | | | | |
| PanR0610 | S [6] | R [6] | S [6] | R [6] | | | | | | | | | | |
| PanR0611 | S [6] | R [6] | S [6] | R [6] | | | | | | | | | | |
| PanR0702 | R [6] | R [6] | R [6] | R [6] | | | | | | | | | | |
| PanR0703 | R [6] | R [6] | R [6] | R [6] | | | | | | | | | | |
| PanR0704 | S [6] | R [6] | S [6] | R [6] | | | | | | | | | | |
| PanR0707 | R [6] | R [6] | R [6] | R [6] | | | | | | | | | | |
| PanR0708 | S [6] | R [6] | S [6] | R [6] | | | | | | | | | | |
| PanR0801 | R [6] | R [6] | S [6] | R [6] | | | | | | | | | | |
| PanR0802 | R [6] | R [6] | R [6] | R [6] | | | | | | | | | | |
| PanR0803 | R [6] | R [6] | S [6] | R [6] | | | | | | | | | | |
| PanR0804 | R [6] | R [6] | S [6] | R [6] | | | | | | | | | | |
| PanR0805 | S [6] | R [6] | S [6] | R [6] | | | | | | | | | | |
| PanR0902 | R [6] | R [6] | R [6] | R [6] | | | | | | | | | | |
| PanR0903 | S [6] | R [6] | S [6] | R [6] | | | | | | | | | | |
| PanR0904 | R [6] | R [6] | R [6] | R [6] | | | | | | | | | | |
| PanR0906 | S [6] | R [6] | S [6] | R [6] | | | | | | | | | | |
| PanR0907 | R [6] | R [6] | R [6] | R [6] | | | | | | | | | | |
| PanR0908 | R [6] | R [6] | R [6] | R [6] | | | | | | | | | | |
| PanR0909 | | R [6] | | R [6] | | | | | | | | | | |
| PanR1005 | R [6] | R [6] | R [6] | R [6] | | | | | | | | | | |
| PanR1006 | R [6] | R [6] | S [6] | R [6] | | | | | | | | | | |
| PanR1007 | S [6] | R [6] | S [6] | R [6] | | | | | | | | | | |
| PanR1101 | | R [6] | | R [6] | | | | | | | | | | |
| R1207 | | R [3] | S [3] | R [3] | | | | | | | | | | |
| R1390 | | S [3] | S [3] | R [3] | | | | | | | | | | |
| R1441 | | S [3] | S [3] | R [3] | | | | | | | | | | |
| R1505 | | R [3] | S [3] | R [3] | | | | | | | | | | |
| R1746 | | R [3] | S [3] | R [3] | | | | | | | | | | |
| R1842 | | S [3] | S [3] | R [3] | | | | | | | | | | |
| R1909 | | R [3] | R [3] | R [3] | | | | | | | | | | |
| RGTB327 | | | | R [11] | | | | | | | | | | |
| RGTB423 | R [11] | | R [11] | R [11] | | | | | | | | | | |
| S96-129 | R : 4 [14] | S [14] | S [14] | R [14] | S [14] | | | S [14] | | S [14] | | | | |
| SP21 | R [15] | R [15] | R [15] | R [15] | R [15] | | R [15] | R [15] | S [15] | S [15] | | | | |
| SUMu001 | S [11] | S [11] | S [11] | S [11] | S [11] | S [11] | S [11] | S [11] | S [11] | S [11] | S [11] | S [11] | S [11] | S [11] |
| SUMu002 | S [11] | S [11] | S [11] | S [11] | S [11] | S [11] | S [11] | S [11] | S [11] | S [11] | S [11] | S [11] | S [11] | S [11] |
| SUMu003 | S [11] | S [11] | S [11] | S [11] | S [11] | S [11] | S [11] | S [11] | S [11] | S [11] | S [11] | S [11] | S [11] | S [11] |
| SUMu004 | S [11] | S [11] | S [11] | S [11] | S [11] | S [11] | S [11] | S [11] | S [11] | S [11] | S [11] | S [11] | S [11] | S [11] |
| SUMu005 | S [11] | S [11] | S [11] | S [11] | S [11] | S [11] | S [11] | S [11] | S [11] | S [11] | S [11] | S [11] | S [11] | S [11] |
| SUMu006 | S [11] | S [11] | S [11] | S [11] | S [11] | S [11] | S [11] | S [11] | S [11] | S [11] | S [11] | S [11] | S [11] | S [11] |
| SUMu007 | S [11] | S [11] | S [11] | S [11] | S [11] | S [11] | S [11] | S [11] | S [11] | S [11] | S [11] | S [11] | S [11] | S [11] |
| SUMu008 | S [11] | S [11] | S [11] | S [11] | S [11] | S [11] | S [11] | S [11] | S [11] | S [11] | S [11] | S [11] | S [11] | S [11] |
| SUMu009 | S [11] | S [11] | S [11] | S [11] | S [11] | S [11] | S [11] | S [11] | S [11] | S [11] | S [11] | S [11] | S [11] | S [11] |
| SUMu010 | S [11] | S [11] | S [11] | S [11] | S [11] | S [11] | S [11] | S [11] | S [11] | S [11] | S [11] | S [11] | S [11] | S [11] |
| SUMu011 | S [11] | S [11] | S [11] | S [11] | S [11] | S [11] | S [11] | S [11] | S [11] | S [11] | S [11] | S [11] | S [11] | S [11] |
| SUMu012 | S [11] | S [11] | S [11] | S [11] | S [11] | S [11] | S [11] | S [11] | S [11] | S [11] | S [11] | S [11] | S [11] | S [11] |
| T17 | S [11] | S [11] | S [11] | S [11] | S [11] | S [11] | S [11] | S [11] | S [11] | S [11] | S [11] | S [11] | S [11] | S [11] |
| T46 | S [11] | S [11] | S [11] | S [11] | S [11] | S [11] | S [11] | S [11] | S [11] | S [11] | S [11] | S [11] | S [11] | S [11] |
| T92 | S [11] | S [11] | S [11] | S [11] | S [11] | S [11] | S [11] | S [11] | S [11] | S [11] | S [11] | S [11] | S [11] | S [11] |
| UM 1072388579 | R [9] | R [9] | S [9] | R [9] | R [9] | R [9] | R [9] | R [9] | R [9] | | | R [9] | S [9] | |
| W-148 | | R [2] | | R [2] | | | | | | | | | | |
| WX1 | R [22] | R [22] | R [22] | R [22] | R [22] | S [22] | S [22] | S [22] | | | | | | |
| WX3 | R [22] | R [22] | R [22] | R [22] | R [22] | S [22] | S [22] | S [22] | | | | | | |
| X122 | | R [3] | S [3] | R [3] | R [3] | | | S [3] | S [3] | | | | | |
| X132 | R [3] | R [3] | S [3] | R [3] | S [3] | R [3] | R [3] | R [3] | S [3] | | | | | |
| X156 | R [3] | R [3] | S [3] | R [3] | S [3] | R [3] | R [3] | R [3] | S [3] | | | | | |
| X189 | S [3] | R [3] | | R [3] | R [3] | R [3] | R [3] | R [3] | S [3] | | | | | |
| X28 | R [3] | R [3] | S [3] | R [3] | R [3] | R [3] | R [3] | R [3] | S [3] | | | | | |
| X29 | R [3] | R [3] | S [3] | R [3] | S [3] | R [3] | S [3] | R [3] | S [3] | | | | | |
| X85 | | R [3] | S [3] | R [3] | R [3] | R [3] | S [3] | R [3] | R [3] | | | | | |
| XDR1219 | R [22] | R [22] | R [22] | R [22] | R [22] | R [22] | R [22] | R [22] | | | | | | |
| XDR1221 | R [22] | R [22] | R [22] | R [22] | R [22] | R [22] | R [22] | R [22] | | | | | | |

## References


1. Elena N. Ilina, Egor A. Shitikov, Larisa N. Ikryannikova, Dmitry G. Alekseev, Dmitri E. Kamashev, Maja V. Malakhova, Tatjana V. Parfenova, Maxim V. Afanasev, Dmitry S. Ischenko, Nikolai A. Bazaleev, Tatjana G. Smirnova, Elena E. Larionova, Larisa N. Chernousova, Alexey V. Beletsky, Andrei V. Mardanov, Nikolai V. Ravin, Konstantin G. Skryabin, and Vadim M. Govorun. Comparative genomic analysis of mycobacterium tuberculosis drug resistant strains from russia. *PLoS ONE*, 8(2):e56577, February 2013.
2. Broad Institute. Broad institute tuberculosis.
3. Thomas R. Ioerger, Yicheng Feng, Xiaohua Chen, Karen M. Dobos, Thomas C. Victor, Elizabeth M. Streicher, Robin M. Warren, Nicolaas C. Gey van Pittius, Paul D. Van Helden, and James C. Sacchettini. The non-clonality of drug resistance in beijing-genotype isolates of mycobacterium tuberculosis from the western cape of south africa. *BMC Genomics*, 11(1):670, November 2010. PMID: 21110864.
4. Thomas R. Ioerger, Sunwoo Koo, Eun-Gyu No, Xiaohua Chen, Michelle H. Larsen, Jr. Jacobs, William R., Manormoney Pillay, A. Willem Sturm, and James C. Sacchettini. Genome analysis of multi- and extensively-drug-resistant tuberculosis from KwaZulu-Natal, south africa. *PLoS ONE*, 4(11):e7778, November 2009.
5. U. Kairov, S. Rakhimova, U. Kozhamkulov, A. Askapuli, M. Zhabagin, V. Bismilda, L. Chingissova, and A. Akilzhanova. Whole genome sequencing of m. tuberculosis in kazakhstan: preliminary data. 2013.
6. Fedora Lanzas, Petros C Karakousis, James C Sacchettini, and Thomas R Ioerger. Multidrug-resistant tuberculosis in panama is driven by clonal expansion of a multidrug-resistant mycobacterium tuberculosis strain related to the KZN extensively drug-resistant m. tuberculosis strain from south africa. *Journal of clinical microbiology*, 51(10):3277–3285, October 2013. PMID: 23884993 PMCID: PMC3811646.
7. Nan Lin, Zhangyi Liu, Jie Zhou, Shihua Wang, and Joy Fleming. Draft genome sequences of two super-extensively drug-resistant isolates of mycobacterium tuberculosis from china. *FEMS Microbiology Letters*, 347(2):9396, 2013.
8. Courtney E. Maus, Bonnie B. Plikaytis, and Thomas M. Shinnick. Molecular analysis of cross-resistance to capreomycin, kanamycin, amikacin, and viomycin in mycobacterium tuberculosis. *Antimicrobial Agents and Chemotherapy*, 49(8):3192–3197, August 2005. PMID: 16048924.
9. Kee Peng Ng, Su Mei Yew, Chai Ling Chan, Jennifer Chong, Soo Nee Tang, Tuck Soon Soo-Hoo, Shiang Ling Na, Hamimah Hassan, Yun Fong Ngeow, Chee Choong Hoh, Kok Wei Lee, and Wai Yan Yee. Draft genome sequence of the first isolate of extensively drug-resistant (XDR) mycobacterium tuberculosis in malaysia. *Genome Announcements*, 1(1):e00056–12, February 2013. PMID: 23405310.
10. D J Ordway, M G Sonnenberg, S A Donahue, J T Belisle, and I M Orme. Drug-resistant strains of mycobacterium tuberculosis exhibit a range of virulence for mice. *Infection and Immunity*, 63(2):741–743, February 1995. PMID: 7822053 PMCID: PMC173064.
11. Nuno S. Osrio, Fernando Rodrigues, Sebastien Gagneux, Jorge Pedrosa, Marta Pinto-Carb, Antnio G. Castro, Douglas Young, Iaki Comas, and Margarida Saraiva. Evidence for diversifying selection in a set of mycobacterium tuberculosis genes in response to antibiotic- and nonantibiotic-related pressure. *Molecular Biology and Evolution*, page mst038, February 2013. PMID: 23449927.
12. Andreas Roetzer, Roland Diel, Thomas A. Kohl, Christian Rckert, Ulrich Nbel, Jochen Blom, Thierry Wirth, Sebastian Jaenicke, Sieglinde Schuback, Sabine Rsch-Gerdes, Philip Supply, Jrn Kalinowski, and Stefan Niemann. Whole genome sequencing versus traditional genotyping for investigation of a mycobacterium tuberculosis outbreak: A longitudinal molecular epidemiological study. *PLoS Med*, 10(2):e1001387, February 2013.
13. Hassan Safi, Brendan Sayers, Manzour H. Hazbon, and David Alland. Transfer of embB codon 306 mutations into clinical mycobacterium tuberculosis strains alters susceptibility to ethambutol, isoniazid, and rifampin. *Antimicrobial Agents and Chemotherapy*, 52(6):2027–2034, June 2008. PMID: 18378710 PMCID: PMC2415778.
14. Linus Sandegren, Ramona Groenheit, Tuija Koivula, Solomon Ghebremichael, Abdolreza Advani, Elsie Castro, Alexandra Pennhag, Sven Hoffner, Jolanta Mazurek, Andrzej Pawlowski, Boris Kan, Judith Bruchfeld, jar Melefors, and Gunilla Kllenius. Genomic stability over 9 years of an isoniazid resistant mycobacterium tuberculosis outbreak strain in sweden. *PLoS ONE*, 6(1):e16647, January 2011.
15. Egor A. Shitikov, Julia A. Bespyatykh, Dmitry S. Ischenko, Dmitry G. Alexeev, Irina Y. Karpova, Elena S. Kostryukova, Yulia D. Isaeva, Elena Y. Nosova, Igor V. Mokrousov, Anna A. Vyazovaya, Olga V. Narvskaya, Boris I. Vishnevsky, Tatiana F. Otten, Valery Y. Zhuravlev, Peter K. Yablonsky, Elena N. Ilina, and Vadim M. Govorun. Unusual large-scale chromosomal rearrangements in mycobacterium tuberculosis beijing B0/W148 cluster isolates. *PLoS ONE*, 9(1):e84971, January 2014.
16. Yoshitaka Tateishi, Aki Tamaru, Yoshitoshi Ogura, Mamiko Niki, Takayuki Wada, Taro Yamamoto, Kazuto Hirata, Tetsuya Hayashi, and Sohkichi Matsumoto. Whole-genome sequence of the potentially hypertransmissible multidrug-resistant mycobacterium tuberculosis beijing strain OM-V02_005. *Genome Announcements*, 1(4):e00608–13, August 2013. PMID: 23929492.
17. SKW Tsui, RCY Chan, KM Kam, HS Kwan, KS Leung, KP Fung, MMY Waye, PTW Law, SK Lou, and KK Leung. Genome analysis of mycobacterium tuberculosis beijing family strains. *Research Fund for the Control of Infectious Diseases*, 19(5), 2013.
18. Shamsudheen Karuthedath Vellarikkal, Ashok Patowary, Meghna Singh, Vinita Periwal, Ajay Vir Singh, Pravin Kumar Singh, Parul Garg, Viswa Mohan Katoch, Kiran Katoch, Pramod Kumar Jangir, Rakesh Sharma, D. S. Chauhan, Vinod Scaria, and Sridhar Sivasubbu. Draft genome sequence of a clinical isolate of multidrug-resistant mycobacterium tuberculosis east african indian strain OSDD271. *Genome Announcements*, 1(4):e00541–13, August 2013. PMID: 23908284.
19. Shamsudheen Karuthedath Vellarikkal, Ajay Vir Singh, Pravin Kumar Singh, Parul Garg, Viswa Mohan Katoch, Kiran Katoch, D. S. Chauhan, Vinod Scaria, and Sridhar Sivasubbu. Draft genome sequence of an extensively drug-resistant mycobacterium tuberculosis clinical isolate of the ural strain OSDD493. *Genome Announcements*, 1(6):e00928–13, December 2013. PMID: 24201204.
20. Shamsudheen Karuthedath Vellarikkal, Ajay Vir Singh, Pravin Kumar Singh, Parul Garg, Viswa Mohan Katoch, Kiran Katoch, D. S. Chauhan, Sridhar Sivasubbu, and Vinod Scaria. Draft genome sequence of a multidrug-resistant clinical isolate of mycobacterium tuberculosis belonging to a novel spoligotype. *Genome Announcements*, 1(6), November 2013. PMID: 24265496 PMCID: PMC3837177.
21. Shamsudheen Karuthedath Vellarikkal, Ajay Vir Singh, Pravin Kumar Singh, Parul Garg, Viswa Mohan Katoch, Kiran Katoch, D. S. Chauhan, Sridhar Sivasubbu, and Vinod Scaria. Draft genome sequence of multidrug-resistant mycobacterium tuberculosis clinical isolate OSDD515, belonging to the uganda i genotype. *Genome Announcements*, 1(6):e00750–13, December 2013. PMID: 24265488.
22. Wei Wu, Huajun Zheng, Lu Zhang, Zilu Wen, Shulin Zhang, Hao Pei, Guohua Yu, Yongqiang Zhu, Zhenling Cui, Zhongyi Hu, Honghai Wang, and Yao Li. A genome-wide analysis of multidrug-resistant and extensively drug-resistant strains of mycobacterium tuberculosis beijing genotype. *Molecular Genetics and Genomics*, 288(9):425–436, September 2013.
23. Yuanyuan Zhang, Chen Chen, Jie Liu, Haijun Deng, Aizhen Pan, Lishui Zhang, Xiuqin Zhao, Mingxiang Huang, Bing Lu, Haiyan Dong, Pengcheng Du, Weijun Chen, and KangLin Wan. Complete genome sequences of mycobacterium tuberculosis strains CCDC5079 and CCDC5080, which belong to the beijing family. *Journal of Bacteriology*, 193(19):5591–5592, October 2011. PMID: 21914894.
