# Supplementary material for: GWAMAR: Genome-wide assessment of mutations associated with drug resistance in bacteria
Source: BMC Genomics. 2014 Dec 12;15(Suppl 10):S10. doi: 10.1186/1471-2164-15-S10-S10 (PMC4304204; doi:10.1186/1471-2164-15-S10-S10)
Supplement: Additional file 2 — Phylogenetic tree for 173 strains of M. tuberculosis. Phylogenetic tree reconstructed using the maximum-likelihood method implemented in the PHYLIP for 173 strains of M. tuberculosis. [file 1471-2164-15-S10-S10-S2.pdf]

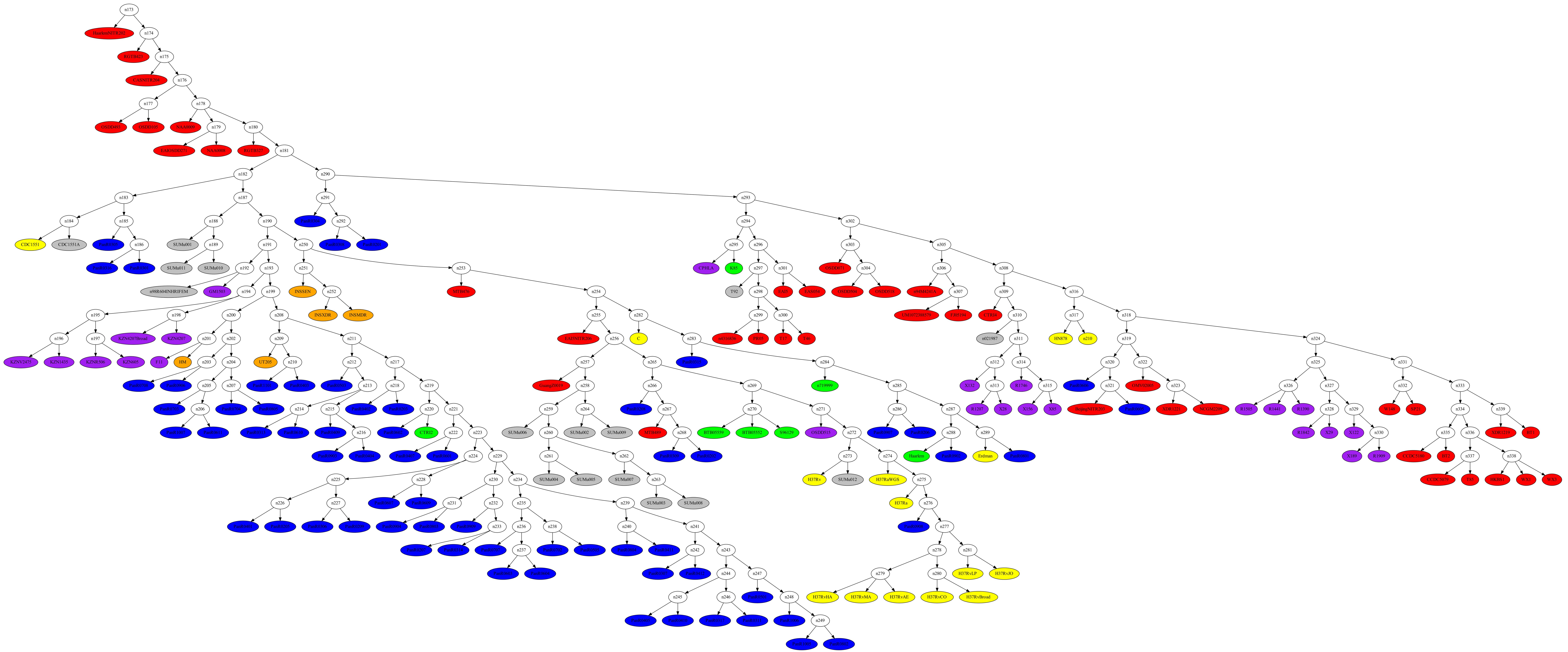

CDC1551
CDC1551A
SUMu001
SUMu011
SUMu010
n98R604INHRIFEM
GM1503
INSSEN
INSXDR
INSMDR
KZN4207Broad
KZN4207
KZNV2475
KZN1435
KZNR506
KZN605
F11
HM
UT205
CTRI2
SUMu006
SUMu002
SUMu009
SUMu004
SUMu005
SUMu007
SUMu003
SUMu008
C
n719999
BTB05559
BTB05552
S96129
OSDD515
H37Rv
SUMu012
H37RaWGS
H37Ra
Haarlem
Erdman
H37RvLP
H37RvJO
H37RvHA
H37RvMA
H37RvAE
H37RvCO
H37RvBroad
CPHLA
K85
T92
n021987
HN878
n210
X132
R1207
X28
R1746
X156
X85
R1505
R1441
R1390
R1842
X29
X122
X189
R1909
