# Supplementary material for: GWAMAR: Genome-wide assessment of mutations associated with drug resistance in bacteria
Source: BMC Genomics. 2014 Dec 12;15(Suppl 10):S10. doi: 10.1186/1471-2164-15-S10-S10 (PMC4304204; doi:10.1186/1471-2164-15-S10-S10)
Supplement: Additional file 3 — Putative compensatory mutations. Complete list of putative compensatory mutations identified in three different studies and our two datasets. [file 1471-2164-15-S10-S10-S3.pdf]

position	ref aa	comas whole-genome 2012	de vos putative 2013	casali evolution 2014	mtu173	mtu broad
10	S				G	
11	E				R	
12	D				T	
13	V				S	
14	L				S	
15	T				P	
16	D				N	
31	G			A,S	S,A	
57	D		N			
165	S		I			
177	K			M		
181	T	I		A		
182	R	L				
183	V	A,G		G		
184	E			D		
186	R	C				
187	T	A:3,P		A,P		
189	A	E				
190	D		G	G		
304	L			R		
307	S			L		
319	E		K			
42	L			F		
45	P			S	L	L,S,A,T
82	E			G		
92	D					G
163	N					K
167	R					C
168	V					G
170	V					F,A
172	Q					R
219	R					C
225	R					G
240	E					G
250	E				G	G
270	D					E
280	P					L
399	T			A		I,A
400	T					A,P,N
401	Q					R
409	Q					R
428	S					R
429	Q					P,H
430	L				P	R,P
431	S					G
432	Q					P,H,E,K,L
434	M					I
435	D				F,V,Y,G,A	Y,V,H,G,A,N
437	N					H,K
438	N					H
439	P					S
441	S					L,*
445	H				D,Y,R	P,D,R,Y,L,N,Q
448	R					Q
450	L					S,W,*
450	S				L	
452	L				P	P,V
454	P					R,L
479	P			T		
480	I					V
481	E					A
482	T					N
483	P					S
487	N				S	
488	I			V		V,L
491	I			V	L	V,F,S,L
494	L					P
496	V			L,M		G,M
503	F			S		S
534	V					G,A
559	A					V
562	V					M
563	E					A
571	D			A		
577	P					A
578	R					H
581	V					L
584	A					D
585	T					A
631	D					G
655	M					T
672	S				Y	
676	T					A
692	A				T	T
693	G					D
695	V					L
700	P					L
702	T					I
723	H			Y		
731	L			P	P	P
741	L					F
761	E			D		D
799	K					Q
827	R			C		C
832	K					E
835	H			P,R	R	R,P
836	G					S
862	L					R
871	R					H
874	S					Y
891	K					E
896	E					K
920	M					V
936	S					R
944	K					E
957	L					V,P
970	V					A
971	F					L
980	Q					R
1012	P					L
1035	I					T
1095	K					R
1106	I				T	
1165	S					F,C
1166	A					V,T
19	D		E			
172	A				V	
332	G		R:2	C,R,S	S	
416	N			T,S		
428	S			A		
431	V			M	M	
432	V	A				
433	G	S		C,S	S	
434	P	R,A		Q,R		
435	Q	H				
445	K			R		
449	L	V		V		
452	F		C	C		
483	V	G:3,A:3	A,G:3	A,G	A,G	
484	W	G:2		G	G	
485	D	N,H	Y	N,Y		
491	I	V:2,T	T:2	V,T	T	
507	L				V	
516	L	P:2		P		
517	L	V:2				
517	V			L		
519	G	D		D	V	
521	A	D		D		
523	G		E			
523	Q	E				
525	H	N	Q	Q		
527	L	V		V	V	
572	R			H		
594	G				E	
601	P				L	
678	P			R		
698	N	S,K,H	H,S	H,K,S	K	
702	E	K				
734	A			V	V	
747	D			A		
761	Q			R		
770	R			H		
826	N			K		
832	I			V		
838	S			C		
847	L			R		
849	Y			C		
901	L		M			
943	D			G		
945	G			V		
989	V				I	
1040	P		R	T,S,R		
1046	I			M		
1071	G		S			
1098	V		G			
1125	Q		H:2			
1133	H				Y	
1150	H				N,P	
1152	K				Q	
1208	M				D,T,X,Y	
1209	G				A,X	
1210	I				S,H,X,P	
1211	T				R,E,X	
1212	K				R,X	
1213	A				R,G,N,X	
1214	S				R,V,X,P	
1215	L				W,A,X	
1216	A				P,G,X,H	
1217	T				P,H,X,R	
1218	D				T,R,X,L	
1219	S				R,L,X,V	
1220	W				G,V,C,X,A	
1221	L				C,A,V	
1222	S				X,R,V,F,G	
1223	A				R,G	
1224	A				X,R,G,V,I	
1225	S				X,R,V	
1226	F				X,S,V,P,L	
1227	Q				X,P,R,G	
1228	E				X,G,R,D,M	
1229	T				D,P,H,A,N,S	
1231	R				C,S	
1240	C				S,L	
1241	R				A	
1242	S				A,C	
1252	V			L	M	
